# Association of waist circumference with blood pressure and familial dietary habits in preschool children: a cross-sectional study in northeastern China

**DOI:** 10.1186/s13052-022-01236-3

**Published:** 2022-04-01

**Authors:** Xiao Tang, Yang Liu, Jiajin Hu, Lingling Zhai, Lihong Jia, Ning Ding, Yanan Ma, Deliang Wen

**Affiliations:** 1 Health Sciences Institute, China Medical University, Shenyang, Liaoning Province 110122 P.R. China; 2 School of Public Health, Dalian Medical University, Dalian, Liaoning Province 116044 P.R. China; 3 Liaoning Key Laboratory of Obesity and Glucose/Lipid Associated Metabolic Diseases, China Medical University, Shenyang, Liaoning Province 110122 P.R. China; 4 School of Public Health, China Medical University, Shenyang, Liaoning Province 110122 P.R. China; 5 Institute of International Medical Education, China Medical University, Shenyang, Liaoning Province 110122 P.R. China

**Keywords:** Blood pressure, Waist circumference, Waist-to-height ratio, Body mass index, Diet

## Abstract

**Background:**

Childhood obesity increases the risk of elevated blood pressure (BP) in children. Body mass index (BMI), waist circumference (WC) and waist-to-height ratio (WHtR) are traditional obesity indices, but the extent to which these indices are associated with elevated BP in childhood remains debatable. Moreover, the familial dietary environment plays an important role in obesity, so it is necessary to determine the most relevant dietary factors for childhood obesity to prevent elevated BP. Our study aimed to identify the obesity indices that are most closely associated with elevated BP and then to determine the independent familial dietary factors for those obesity indices.

**Method:**

A total of 605 children aged 2 to 6 years, as well as their parents, were involved in this study. The weight, height, WC and BP of the children were measured. Information on familial environments was obtained by questionnaires completed by the parents. BMI, WC and WHtR were standardized into z scores, and categorical variables of these three obesity indices were defined as BMI Category, WC Category and WHtR Category. Logistic regression was used to analyse the associations between all obesity indices and elevated BP. Multivariate linear regression and logistic regression were used to determine the independent factors for obesity indices.

**Results:**

The obesity indices that were most closely associated with elevated BP were WC and WC Category. Parental BMI, birth weight, eating wheat as a staple food, appetite, eating speed, snacking while watching TV, parental encouragement to eat a diverse assortment of foods and drinking milk were independently associated with WC in both males and females. The risk of abdominal obesity increased 1.375 times in males and 1.631 times in females if appetite increased one level. If eating speed increased one level, the risk of abdominal obesity increased 1.165 times in males and 0.905 times in females. Females who drank milk more than 6 times per week had a 0.546 times lower risk of abdominal obesity.

**Conclusion:**

WC was an anthropometric parameter more closely associated with elevated BP. In addition to genetics, some familial dietary factors involving eating preference, eating habits and parental feeding practice were independently associated with WC and abdominal obesity in preschool children.

**Supplementary Information:**

The online version contains supplementary material available at 10.1186/s13052-022-01236-3.

## Background

Hypertension is a common chronic disease in modern society. There is an abundance of evidence suggesting that elevated blood pressure (BP) during childhood can lead to negative outcomes, such as vascular damage, cardiometabolic risk and organ damage in childhood [1–3], and it can increase the individual’s risk of hypertension in adulthood [2, 4, 5]. Preventing elevated BP in children is beneficial for controlling the prevalence of hypertension in adults, which can in turn decrease adult mortality due to cardiovascular diseases [6, 7]. However, symptoms of elevated BP in children are not apparent, and the criteria for screening and diagnosis are not consistent [1], which leads to underestimation of the prevalence of elevated BP in children [8].

Obesity has long been recognized as an important cause of elevated BP in children [9–12]. Recent studies have attempted to determine the best marker of elevated BP among anthropometric parameters such as body mass index (BMI), waist circumference (WC) and waist-to-height ratio (WHtR). BMI is the most common index used to predict elevated BP [11, 13]. However, BMI cannot fully express the distribution of an individual’s body fat. WC is commonly used to describe abdominal obesity. Some studies have shown that WC is strongly associated with elevated BP during childhood, as well as other cardiovascular diseases in adulthood [14, 15]. WC has also become one of many indices of metabolic abnormalities among adults. WHtR is a waist circumference-related index that considers height. Some studies have pointed out that WHtR plays an important role in predicting elevated BP or risk of cardiovascular diseases during childhood [16, 17]. However, the result on the magnitudes of associations of BMI, WC and WHtR with elevated BP in childhood is inconclusive. Some studies have suggested that BMI, WC and WHtR have no differences in predicting childhood hypertension, whereas others have insisted on the difference between these indictors [10, 18]. In addition, in previous studies investigating the relationship between obesity indices and elevated BP in children, the indices analysed were either only numerical variables or only categorical variables [10, 16–18]. This may be one of the reasons for the inconsistency. Considering WC, BMI, WHtR and their categorical indicators, determining which of these adiposity indicators, is most strongly associated with elevated BP in children needs further investigation. Moreover, most research has studied school-age children and adolescents [9–11], and studies on preschool children are very limited [12].

Since obesity is thought to cause elevated BP, it is necessary to explore the risk factors for obesity among preschool children. Many studies have shown that the familial dietary environment, including dietary patterns, eating behaviours and parental feeding practices, has a large impact on a child’s weight [19–22]. With respect to dietary patterns, China has experienced rapid economic and social development over three decades, and a major change in food consumption and food components has occurred. Dietary patterns have transitioned from traditional to Westernized patterns. People have reduced the consumption of vegetables and whole grains and increased the consumption of red meat, processed meat, refined grain, sugar-sweetened beverages, saturated fat and fried food, which has led to an increase in the prevalence of obesity, diabetes and cardiovascular disease [23, 24]. In addition, people in northeastern China prefer the typical northern Chinese diet (eating root vegetables, foods high in sugar and fat, and wheat and wheat-based products as a staple food) and the modern diet (high intake of milk, fast food and eggs). These two dietary patterns are more likely to cause obesity than the dietary pattern in southern China (high intake of vegetables, rice and pork) [25].

There is strong evidence that eating behaviours play an important role in childhood obesity. Many studies have investigated the association of obesity with eating behaviours [26, 27]. Most of these studies found that some eating behaviours, such as higher responsiveness to internal satiety cues and slowness in eating, are negatively correlated with obesity, whereas other eating behaviours, such as eating in response to environmental food cues and food enjoyment, are positively correlated with obesity [22, 23]. In addition, unhealthy eating behaviours such as eating quickly, eating while watching TV and snacking while watching TV have been reported as factors contributing to childhood obesity [27–29]. Previous studies have claimed that a fast eating speed interferes with the reflex signal that tells the body to stop eating when the stomach is full, which leads to more food intake but lower satiety [28]. Eating or snacking while watching TV increases energy intake due to overeating in front of the TV [30].

Parents can have direct control over their child’s diet, so the familial dietary environment is also influenced by parental restrictions and guidance. Parental feeding practices reflect the parents’ attitudes towards nutritional science. The most important nutrition-related parenting practices include limiting certain types of food, encouraging or asking children to eat a diverse range of foods, using food as a reward and ensuring food availability at home [26, 27, 30]. As demonstrated in many studies, parental restrictions and guidance on diet determine the amount and type of foods the child ingests and therefore are associated with the child’s weight [31, 32]. An uninvolved, highly protective or strict parenting style is generally associated with higher BMI in children, while authoritative parenting is associated with healthy BMI [31, 32].

Although there are many studies analysing the relationship between familial dietary environments and childhood obesity, most of the studies have only focused on one aspect of diet, such as eating behaviours, dietary patterns or parental feeding practices. However, among the numerous factors in multiple dimensions, it is unclear which familial dietary factors are independent factors for childhood obesity. Our study first compared the magnitude of associations between BMI, WC, WHtR and elevated BP in preschool children. Then, we determined independent familial dietary factors for obesity indices that were the most strongly associated with elevated BP, offering important variables to target in preventing elevated BP among preschool children.

## Method

### Participants

The participants of this study were preschool children aged 2-6 years in Shenyang, China. Multistage sampling was used to select the sample. From the seven districts in Shenyang, four were randomly selected. From each of the four districts, two kindergartens were randomly selected. Convenience sampling was employed to select one class from each grade in each kindergarten. The eligibility criteria for participating included the following: (1) the parents’ ethnicity was Chinese; (2) the children were between 2 and 6 years old at the time of enrolment; and (3) the parents of these children could read and write. A total of 635 children and their parents were recruited. Physical examinations were performed on these children by trained nurses, and their parents were asked to complete the questionnaires at home. Children were excluded from the study if (1) they had developmental diseases; (2) their parents refused to give consent; or (3) their parents could not complete the questionnaires. Finally, 605 children and their parents participated in this study.

This study was approved by the Ethics Committee of the Institute of Health Science (Ethics Approval No. [2017] 055), China Medical University.

### Measurements

#### Dependent variable

The dependent variable of the first aim of the study was BP. The physical examinations were conducted by trained and licenced doctors during school hours. The children rested for at least 5 min before their blood pressures were measured. Blood pressure was measured by using an auscultation mercury sphygmomanometer with an appropriate cuff size. The systolic pressure was recorded as the pressure when the sphygmomanometer made the first Korotkoff sound. The diastolic pressure was taken when the sphygmomanometer made the fifth Korotkoff sound. BP was measured twice for each child, and the average was taken. Based on the sex- and age-specific distribution of Chinese children’s blood pressures [33], elevated BP was defined as either systolic or diastolic pressure above the 90th percentile, and normal BP was defined as both systolic and diastolic pressures below the 90th percentile. A discrete variable was created with “normal BP” being the reference group (0 = normal BP and 1 = elevated BP).

The dependent variables of the second aim of the study were obesity indices. Heights and weights were measured with the children standing up, barefoot and wearing undergarments. BMI was calculated as weight (kg) divided by the square of height (m). According to the sex- and age-specific BMI cut-off points provided by the Obesity Working Group, China (OWGC) [34], subjects were classified into one of three categories: healthy weight, overweight or obese. We created a discrete variable, “BMI Category” (0 = healthy weight, 1 = overweight or obesity).

WC was measured by wrapping a nonelastic flexible measuring tape around children’s waists 1 cm above the navel. The measurements were taken at normal expiration. Children whose WC was above the sex- and age-specific 80th percentile provided by OWGC were determined to be abdominally obese [35]. We created a discrete variable, “WC Category” (0 = not abdominally obese, 1 = abdominally obese).

WHtR was calculated as waist circumference (cm) divided by height (cm). Children whose WHtR was greater than or equal to 0.5 were determined to be abdominally obese. The variable “WHtR Category” was created as a discrete variable (0 if WHtR was less than 0.5 and 1 if WHtR was greater than or equal to 0.5).

Because males and females have different distributions of BMI, WC and WHtR values, we standardized these three obesity indices. Based on the sex- and age-specific averages and standard deviations of BMI, WC and WHtR provided by OWGC, BMI, WC and WHtR values were standardized into z score values ZBMI, ZWC, and ZWHtR, respectively [34, 35]. Standardized z score values for BMI, WC and WHtR were all calculated as:

$$z=\frac{value- mean}{Standard\ deviation}$$

#### Questionnaires and independent variables

A self-designed questionnaire was used, and the parents were asked to complete the questionnaire at home. The questionnaire asked about children’s and parents’ demographic information, children’s food preferences, children’s eating behaviours, parental control of children’s diets, children’s sleep duration and children’s levels of physical activity.

Demographic information included the child’s age and gender, and parents’ heights, weights, levels of education and familial income. Paternal BMI and maternal BMI were calculated. Familial income level was recorded as < 3000 yuan, 3000 to 5000, 5000 to 8000, and > 8000. Parents’ education was categorized into three levels: secondary school or less, college or bachelor’s degree, and graduate degree. The child’s age, paternal BMI and maternal BMI were used as continuous variables, and familial income level and parents’ education were used as dummy variables in the regression models.

Familial dietary information included the children’s food preferences, eating behaviours and parental control and guidance on the children’s diets. For the child’s food preferences, parents were asked whether their child normally eats wheat and wheat-based products as their staple food (0 = no, 1 = yes). They also reported how many times a week their child eats deep-fried food; potatoes and other root vegetables; milk products; desserts and sweets; nuts; and puffed food. The child’s eating behaviours included eating speed (1 = slow, 2 = average, 3 = fast), watching TV while eating (0 = yes, 1 = no), snacking while watching TV (0 = yes, 1 = no) and appetite (1 = good, 2 = average, 3 = poor). Parental control and guidance on the children’s diets included whether they limit the amounts of snacks their child eats (0 = yes, 1 = no), whether they use food as rewards (0 = yes, 1 = no), and whether they encourage their child to eat a diverse assortment of foods (0 = yes, 1 = no).

Sleep duration was the amount of sleep the child gets during the night combined with the amount of sleep the child gets during the day (in hours). The amount of physical activity the child gets was the total amount of moderate- and vigorous-intensity physical activity per day (in hours) which can make the child gasp, such as brisk walking, running, jumping, bike riding, dancing and climbing.

### Statistical analysis

Numerical variables with a normal distribution were expressed as the mean and standard deviation, and numerical data with a skewed distribution were expressed as percentiles. Categorical variables were shown as frequencies and percentages. The difference in ZWC between the elevated BP and normal BP groups was analysed by two-sample t-tests, and the differences in ZBMI and ZWHtR between these two groups were analysed by the Wilcoxon Mann–Whitney test. The differences in the prevalence of overweight and abdominal obesity between the elevated and normal BP groups were analysed by the chi-squared test. Logistic regression was used to analyse the association between obesity indices (ZBMI, ZWC, ZWHtR, BMI Category, WC Category, WHtR Category) and elevated BP in preschool children. Elevated BP was the outcome variable in the logistic regression, and five models were constructed. Each model included two different obesity indices that were both numerical or both categorical to compare the sensitivity of the indices to elevated BP. All models were adjusted by age and amount of physical activity.

To investigate the relationship between familial factors and the obesity indices, ZWC and WC category were used as dependent variables in the linear regression and logistic regression, respectively. First, each independent variable was entered in the univariate regression, and variables that yielded a P value less than 0.2 remained. Second, a multivariate stepwise linear regression and a multivariate stepwise logistic regression containing independent variables remaining from the previous step were then performed to find the independent factors for the ZWC and WC Category, respectively.

Excel 17.0 was used to record the collected data. SPSS 22.0 was used for statistical analysis.

## Results

The basic characteristics of the participating children are shown in Table 1. Approximately 14.0% of the children were overweight, and 36.0% of the children were obese. A total of 50.7% of the children had a WHtR ≥0.5, and 15.9% had elevated BP. Approximately half of the parents asked their children to eat a diverse assortment of foods. Approximately 40% of parents rewarded their children with food. Approximately 80% of parents limited the amounts of snacks their children ingested. More than 40% of the children had the habit of eating their meals while watching TV or snacking while watching TV. The values of all variables were not significantly different between male and female children. The results suggest that dietary preferences, dietary habits and whether the parents exerted control over the children’s diets had no significant differences between male and female children.

**Table 1:** The basic characteristics of participating children

	Males (*n* = 384)	Females (*n* = 221)	*P*
**Children’s characteristics**			
Age (years), $\overline{\mathrm{X}}\left(\mathrm{S}\right)$	4.4 (1.0)	4.4 (1.2)	0.919
Birth weight (kg), $\overline{\mathrm{X}}\left(\mathrm{S}\right)$	3.5 (0.5)	3.4 (0.4)	0.652
**Anthropometric and blood pressure measurements, median (P**_**25**_**, P**_**75**_**)**			
Height (cm)	111.4 (105.0, 118.0)	110.5 (102.5, 117.2)	0.227
Weight (kg)	10.4 (8.8, 12.6)	10.1 (8.3, 12.2)	0.094
WC (cm)	54.9 (51.0, 62.0)	55.0 (51.0, 62.0)	0.748
BMI	16.8 (15.4, 19.5)	16.7 (15.4, 19.2)	0.396
WHtR	0.5 (0.5, 0.6)	0.50 (0.5, 0.6)	0.529
DBP (mmHg)	90.0 (85.0, 99.0)	90.0 (84.0, 96.0)	0.273
SBP (mmHg)	60.0 (50.0, 62.0)	60.0 (50.0, 60.0)	0.139
**Weight and blood pressure status,** ***n*** **(%)**			
Healthy weight	188 (49.0)	114 (51.6)	
Overweight	54 (14.1)	31 (14.0)	0.797
Obesity	142 (37.0)	76 (34.4)	
WHtR ≥0.5	197 (51.3)	111 (50.2)	0.841
WC ≥ P_80_	209 (54.5)	132 (59.7)	0.184
Elevated BP	69 (18.0)	38 (17.2)	0.783
**Food preferences,** ***n*** **(%)**			
Puffed foods ≥ once per week	175 (45.7)	107 (48.6)	0.505
Deep-fried foods ≥ twice per week	59 (15.3)	33 (15.1)	0.947
Potatoes and other root vegetables ≥4 times per week	168 (43.8)	94 (42.5)	0.769
Milk ≥6 times per week	179 (46.6)	107 (48.4)	0.729
Nuts ≥3 times per week	299 (77.9)	180 (81.4)	0.365
Sweets ≥3 times per week	101 (26.3)	60 (27.1)	0.82
Eating wheat and wheat products as their staple food	54 (14.1)	36 (16.3)	0.459
**Eating habits,** ***n*** **(%)**			
Appetite			
Good	239 (62.2)	131 (59.3)	
Average	135 (35.2)	77 (34.8)	0.139
Poor	10 (2.6)	13 (5.4)	
Eating speed			
Fast	90 (23.4)	41 (18.5)	
Average	220 (57.3)	123 (55.7)	0.088
Slow	74 (19.3)	57 (25.8)	
Eating while watching TV	167 (43.5)	93 (42.1)	0.648
Snacking while watching TV	142 (37.0)	98 (44.5)	0.067
**Other behaviours**, **median (P**_**25**_**, P**_**75**_**)**			
Sleep (hours)	11.0 (10.1, 12.0)	11.0 (10.0, 12.0)	0.124
Physical activity (hours)	1.0 (1.0, 2.0)	1.0 (1.0, 2.0)	0.601
**Parental characteristics**			
Parental BMI, median (P_25_, P_75_)			
Maternal BMI	22.0 (20.3, 24.1)	22.0 (20.4, 23.9)	0.730
Paternal BMI	25.1 (22.8, 27.8)	25.5 (23.1, 28.0)	0.876
**Familial monthly income (¥),** ***n*** **(%)**			
< 3000	22 (5.7)	20 (9.0)	0.237
3000 to 5000	90 (23.4)	45 (20.4)	
5000 to 8000	130 (33.9)	65 (29.4)	
> 8000	142 (37.0)	91 (41.2)	
**Parental education level,** ***n*** **(%)**			
Secondary school or less	110 (28.7)	54 (24.4)	0.510
College or Bachelor’s degree	240 (62.5)	145 (65.6)	
Graduate degree	34 (8.9)	22 (10.0)	
**Maternal education level,** ***n*** **(%)**			
Secondary school or less	104 (27.1)	54 (24.4)	0.298
College or Bachelor’s degree	242 (63.0)	152 (68.8)	
Graduate degree	38 (9.9)	15 (6.8)	
**Parental control and guidance,** ***n*** **(%)**			
Parental encouragement to eat a diverse range of foods	202 (52.6)	116 (52.5)	0.978
Using food as a reward	137 (35.7)	88 (39.8)	0.434
Limiting the amounts of snacks	324 (84.4)	173 (78.3)	0.103

*SD* standard deviation, *BMI* body-mass index, *WC* waist circumference, *WHtR* wasit-to-height ratio, *DPB* diastolic blood pressure, *SBP* systolic blood pressure, *P* percentile, *BP* blood pressure, *ZBMI* standardized z-score values of BMI, *ZWC* standardized z-score values of WC, *ZWHtR* standardized z-score values of WHtR

*significant at *P* <  0.05

Obesity index differences between the elevated BP and normal BP groups are displayed in Table 2. For male children, those with elevated BP had higher ZWC, ZWHtR and ZBMI than those with normal BP. Among male children, there was a higher prevalence of abdominal obesity (as determined by WHtR and WC) and overweight (as determined by BMI) in those with elevated BP than in those with normal BP. For female children, the prevalence of abdominal obesity (as determined by WC) was higher in those with elevated BP than in those with normal BP.

**Table 2:** Obesity index differences between the elevated BP and normal BP groups

Obesity indices	Males (*n* = 384)			Females (*n* = 221)		
	Elevated BP (*n* = 69)	Normal BP (*n* = 315)	*P*	Elevated BP (*n* = 38)	Normal BP (*n* = 183)	*P*
ZWC, median (P_25_, P_75_)	2.13 (0.69, 3.59)	0.57 (− 0.17, 2.02)	< 0.001**	1.50 (0.85, 2.95)	0.83 (0.2, 2.82)	0.068
ZWHtR, median (P_25_, P_75_)	2.00 (0.19,3.09)	0.48 (− 0.35, 1.85)	< 0.001**	1.42 (0.56,2.39)	1.07 (0.09, 2.75)	0.335
ZBMI, median (P_25_, P_75_)	3.03 (0.55,4.58)	0.90 (0.72, 2.97)	< 0.001**	1.82 (0.32,3.25)	0.93 (−0.01, 2.66)	0.099
Overweight, *n* (%)	49 (71.0)	153 (48.6)	0.001*	23 (60.5)	86 (47.0)	0.161
WHtR ≥0.5, *n* (%)	47 (68.1)	148 (46.9)	0.002*	21 (55.2)	86 (47.0)	0.442
WC ≥ P_80_, *n* (%)	57 (82.6)	161 (51.1)	< 0.001**	31 (81.6)	110 (60.1)	0.019*

*BP* blood pressure, *ZBMI* standardized z-score values for BMI, *ZWC* standardized z-score values for WC, *ZWHtR* standardized z-score values for WHtR

*significant at *P* <  0.05

^**^significant at *P* <  0.001

Univariate logistic regression results showed that for male children, all obesity indices, whether numerical or categorical, were related to elevated BP. For female children, only WC Category was associated with elevated BP (Supplementary Table 1). For the multivariate analysis, BMI-related obesity indices and WC-related obesity indices were combined. The results showed that among male children, when ZWC and ZBMI were included in the model together, ZWC had a stronger association with elevated BP. When ZWHtR and ZBMI were included in the model together, ZBMI had a stronger association with elevated BP (Table 3). Therefore, for male children, numerical obesity indices that had associations with elevated BP were, in decreasing order of strength: ZWC, ZBMI and ZWHtR. When BMI Category and WC Category were put in the model together, WC Category showed a stronger association. When WHtR Category and WC Category were combined in the model, WC Category was significant. When WHtR Category and BMI Category were put in the model together, neither of them was statistically significant. Therefore, compared to BMI Category and WHtR Category, WC Category showed a stronger association with elevated BP in male children. For female children, when WHtR Category and WC Category were combined, WC Category was superior. Although no obesity indices were associated with elevated BP in other models, WC Category was almost significant (*P* = 0.054) when WC Category and BMI Category were analysed together. The results suggested that for female children, WC Category was also the obesity index that had the closest association with elevated BP (Table 3).

**Table 3:** The associations of obesity indices with elevated BP by logistic regressions

Variable	Males		Females	
	OR (95% CI)	*P*	OR (95% CI)	*P*
**Model 1**				
ZBMI	1.06 (0.89, 1.26)	0.549	1.14 (0.87, 1.50)	0.351
ZWC	1.30 (1.03, 1.64)	0.029*	1.06 (0.82, 1.38)	0.667
**Model 2**				
ZBMI	1.27 (1.05, 1.55)	0.016*	1.19 (0.92, 1.53)	0.185
ZWHtR	0.96 (0.75, 1.23)	0.749	0.99 (0.81, 1.23)	0.999
**Model 3**				
BMI Category^a^	1.37 (0.68, 2.76)	0.385	1.11 (0.47, 2.62)	0.811
WC Category^b^	3.64 (1.63, 8.16)	0.002*	2.81 (0.98, 8.03)	0.054
**Model 4**				
BMI Category	1.88 (0.93, 3.84)	0.081	1.69 (0.67, 4.28)	0.269
WHtR Category^c^	1.87 (0.93, 3.77)	0.081	1.02 (0.41, 2.57)	0.963
**Model 5**				
WC Category	4.05 (1.66,9.90)	0.002*	3.68 (1.22,11.07)	0.021*
WHtR Category	1.11 (0.51,2.41)	0.800	0.72 (0.29,1.76)	0.466

All models were adjusted by age and the amounts of physical activity

^a^BMI category: overweight/ obesity verses healthy

^b^WC category: WC ≥ P_80_ versus WC < P_80_

^c^WHtR category: WhtR ≥0.5 versus WHtR < 0.5

^*^significant at *P* <  0.05

Since ZWC and WC Category were the numerical and categorical obesity indices that were most closely associated with elevated BP among the preschool children, respectively, Tables 4 and 5 show the independent factors for these two variables. For male children, parental BMI, fast eating speed, good appetite, snacking while watching TV and eating wheat and wheat-based products as their staple food were positively associated with ZWC. Male children with faster eating speed, better appetite, habits of snacking while watching TV and eating wheat-based products as their staple food had larger ZWCs. For female children, maternal BMI and a good appetite were positively associated with ZWC. For both male and female children, parental encouragement to eat a diverse assortment of foods was negatively associated with ZWC (Table 4).

**Table 4:** The independent factors for ZWC by multivariate stepwise linear regressions

Independent variables	Males		Females	
	B (95% CI)	*P*	B (95% CI)	*P*
Age	0.480 (0.310, 0.650)	< 0.001	0.363 (0.179, 0.546)	< 0.001
Paternal BMI	0.092 (0.047, 0.137)	< 0.001	–	–
Maternal BMI	0.092 (0.040, 0.143)	0.001	0.141 (0.073, 0.209)	< 0.001
Eating wheat and wheat products as their staple food^a^	0.756 (0.270, 1.241)	0.002	–	–
Appetite^b^	0.556 (0.197, 0.914)	0.002	0.870 (0.496, 1.244)	< 0.001
Eating speed^c^	0.571 (0.277, 0.864)	< 0.001	–	–
Parental encouragement to eat a diverse range of foods^d^	−0.677 (−1.081, −0.274)	0.001	−0.700 (−1.144, −0.257)	0.002
Snacking while watching TV^e^	0.373 (0.026,0.721)	0.035	–	–

*BMI* body-mass index

^a^Eating wheat and wheat-based products as their staple food: “yes” versus “no”

^b^The three levels of appetite: poor, average, good, were given the values 1, 2, and 3, respectively

^c^The three eating speeds: slow, average, fast, were given the values of 1, 2 and 3, respectively

^d^Encouraging child to eat a diverse range of foods: “yes” versus “no”

^e^Snacking while watching TV: “yes” versus “no”

**Table 5:** The independent factors for WC Category by multivariate stepwise logistic regressions

Independent variables	Males		Females	
	OR (95% CI)	*P*	OR (95% CI)	*P*
Age	1.653 (1.286, 2.124)	< 0.001	1.445 (1.093, 1.910)	0.010
Birthweight	1.316 (1.001, 1.713)	0.041	1.110 (0.998, 1.234)	0.054
Paternal BMI	1.117 (1.045, 1.194)	0.001	–	–
Maternal BMI	1.093 (1.012, 1.180)	0.024	–	–
Appetite^a^	2.375 (1.443, 3.908)	0.001	2.631 (1.479, 4.682)	0.001
Eating speed^b^	2.265 (1.486, 3.454)	< 0.001	1.905 (1.109, 3.270)	0.019
Milk intake^c^	–	–	0.454 (0.238, 0.866)	0.016

^a^The three levels of appetite: poor, average, good, were given the values 1, 2, and 3, respectively

^b^The three eating speeds: slow, average, fast, were given the values of 1, 2 and 3, respectively

^c^Milk intake: drinking milk ≥6 times per week versus drinking milk < 6 times per week

Table 5 shows that for male children, birth weight, parental BMI, a good appetite and eating speed were positively associated with WC-determined abdominal obesity. For female children, maternal BMI, good appetite and fast eating speed were positively associated with WC-determined abdominal obesity, whereas milk intake was negatively associated with abdominal obesity. The risk of abdominal obesity increased 1.375 times in males and 1.631 times in females if appetite increased one level. If eating speed increased one level, the risk of abdominal obesity increased 1.165 times in males and 0.905 times in females. Children who drank milk at least 6 times per week had a 0.546 times lower risk of abdominal obesity than those who did not.

## Discussion

Our study compared the magnitudes of association between elevated BP and the obesity indices of BMI, WC and WHtR among preschool children aged 2-6. The results suggested that WC and abdominal obesity defined by WC were strongly associated with elevated BP. It was also found that, among familial factors, certain food-related factors have a strong association with WC and abdominal obesity. This conveys what we need to target in preventing obesity and adult hypertension in the future.

In our study, the regression models showed that WC and WC Category showed a stronger positive association with elevated BP than other obesity indices. These results were similar to some previous studies. A study performed on a sample of Brazilian children aged 8 to 10 years confirmed that abdominal obesity determined by WC had the closest association with elevated BP compared to obesity determined by BMI and the percentage of total body fat (PBF) [15]. The risk of hypertension in Mexican children and adolescents was found to be higher in those with increased WC than in those with high BMI [36]. Based on the results of some other studies, whether WC is the best predictor of elevated BP is still under debate. A Chinese national cross-sectional study of children and adolescents aged 9-17 concluded that WHtR had a stronger ability to predict elevated BP after it was adjusted based on BMI values, which was different from our conclusion [37]. A cohort study in China showed that high BMI, but not WC or PBF, was associated with a higher risk of childhood hypertension [9]. However, past studies only analysed either numerical or categorical obesity indices in a single study, never both. In contrast, our research included both types of obesity indices, which may provide a more comprehensive analysis of the association between obesity indices and elevated BP. Our results suggested that WC should be screened to decrease the risk of elevated BP.

In our study, WC and WC Category showed a strong association with elevated BP, so we determined the potential familial factors associated with WC and WC Category to provide a prevention strategy. According to the results, apart from birth weight and parental BMI, all other independent factors were related to dietary preference, eating behaviour and parental feeding style. In the current study, for both male and female children, a good appetite predicted increased WC and an increased risk of abdominal obesity, which is also what previous studies have found [27]. A greater interest in food, a better appetite, enjoyment of food, food responsiveness, desire to drink and emotional overeating have been consistently associated with overweight/obesity in childhood.

In the current study, it seemed that the faster children eat, the greater the waist circumference in males, and the greater the risk of developing abdominal obesity in males and females. Although many studies have claimed a positive relationship between eating speed and general obesity in children, few studies have investigated the association of eating speed with abdominal obesity. A recent study based on a nationwide school-based intervention program of obesity in China showed that eating quickly increased the risk of abdominal obesity defined by WC among school students [38]. This result was consistent with ours. Many previous studies have found that slowness in eating is a type of food avoidance behaviour and is negatively correlated with obesity [27]. There are two possible reasons why eating slowly is good for maintaining a healthy weight. First, eating slowly may enhance the thermic effect of food, which benefits weight control [39]. Second, eating speed may influence gastrointestinal satiety hormones, which can control appetite and influence food intake [40].

In our study, the habit of snacking while watching TV was also found to be an independent factor for an increase in WC in male children. Previous studies have shown that there is a positive relationship between television viewing time and BMI or WC, which might be due to an increase in sedentary behaviour, the power of advertising on food intake and an increase in food intake [41]. Obviously, snacking while watching TV can cause irregular and excess food intake, leading to overweight and obesity. One study claimed that reducing the TV viewing time to 1 h per week could prevent 29% of obesity [42]. Therefore, parents should reduce the amount of time their children spend watching TV and forbid the practice of snacking while watching TV.

Our study also concluded that encouraging children to eat a wide variety of foods was negatively associated with WC, which was similar to the conclusion of a Polish study of 5-year-olds [32]. Eating a diverse range of foods may promote proper physical development and therefore can help in weight control. Early introduction of a diverse assortment of foods can also broaden a child’s food preferences and help the child develop enjoyment of healthy foods. On the other hand, limited exposure to novel foods can lead to solidifying the child’s preferences for unhealthy foods, consequently leading to an elevated risk of being overweight or obese [43, 44].

Our study found that the frequency of milk intake is a protective factor for obesity in girls but not in boys. Some previous studies have also found that milk intake negatively correlates with both body weight and body fat [45–47]. This may be because milk contains calcium, conjugated linoleic acid and milk proteins, which all promote proper development, making milk beneficial for maintaining weight [44]. However, the relationship between milk intake and obesity is still under debate [46, 48]. A systematic review showed that in high-income countries, there is either no association or an inverse association between milk intake and overweight and obesity in preschool children [49]. Another review including twenty randomized controlled trials, thirty-one longitudinal cohort studies and forty-three cross-sectional studies obtained similar results in children and adolescents [50]. Regarding the influence of milk fat content, a review study suggested that higher cow milk fat intake was beneficial for lower childhood adiposity [51]. Some studies also showed that whole milk consumption was associated with a decrease in BMI [45, 46, 52]. Therefore, further research is needed to determine the association between milk intake and childhood obesity. However, major evidence suggests that milk intake does not increase the risk of childhood obesity, so milk intake should be encouraged because it provides children with nutrients for growth.

Our study showed that male children who eat wheat and wheat-based products as their staple food have a larger WC than those who do not. In northern China, wheat and wheat-based products, such as noodles and steamed buns, are common staple foods [19]. The prevalence of obesity is also higher in northern China than in southern China, where the staple food is rice [19, 53]. Compared to rice, wheat absorbs less water when cooked, so it is calorically denser. There is a strong correlation between obesity and the intake of calorically dense foods [54].

In the current study, it was found that parental BMI was strongly associated WC and abdominal obesity in males. A study performed on Beijing children showed that the risk of male children becoming obese is higher if both of their parents are obese than if only one of their parents is obese. In female children, however, the risk of obesity is the same whether both parents are obese or if only one parent is obese [55]. These results are partially similar to what we found. The fact that parental BMI is associated with the child’s weight can be explained by shared genetics and shared environments. Studies have found 50 polymorphic genes related to BMI [56]. With respect to the shared environment, parental habits, such as dietary habits and exercise habits, can influence the child’s habits.

This study has several limitations. First, cross-sectional studies cannot confirm causality, so further cohort studies are required. Second, elevated BP was determined from a single visit but hypertension was not clinically diagnosed. Third, the information on eating patterns was not exhaustive enough in the questionnaire, so the quantity of food could not be assessed. However, the frequency of food intake could partly reflect the habitual eating patterns. Fourth, the questionnaire did not include information on a family history of hypertension that should be considered a factor of familial predisposition. Finally, a larger sample size would have been better.

Our study was based on a sample of preschool children and identified the paramount factors for WC and abdominal obesity that were strongly associated with elevated BP. Research on preschool children is very limited. It is well known that insulin resistance and hyperinsulinaemia are physiological changes in normal pubertal children, while insulin resistance plays an important role in hypertension [1, 57]. Therefore, our study excluded the effect of puberty on insulin resistance, making the relationship between obesity indices and elevated BP clearer in preschool children. This is the main strength of our study.

## Conclusion

WC was an anthropometric parameter more closely associated with elevated BP. Genetics and familial dietary habits are the main predictors of increased WC and abdominal obesity in preschool children. High parental BMI, eating wheat and wheat-based products as a staple food, good appetite, fast eating speed and snacking while watching TV were risk factors for increased WC and abdominal obesity. Parental encouragement to eat a diverse assortment of foods and drinking milk were protective factors.

## Supplementary Information

**Additional file 1: Supplementary Table 1.** Associations of obesity indices with elevated BP by univariate logistic regressions.

## Data Availability

The datasets used and analysed during the current study are available from the corresponding author on reasonable request.

## Abbreviations

BMI: Body mass index
WC: Waist circumference
WHtR: Waist-to-height ratio
PBF: Percentage of total body fat
