# Supplementary material for: Association of waist circumference with blood pressure and familial dietary habits in preschool children: a cross-sectional study in northeastern China
Source: Ital J Pediatr. 2022 Apr 1;48:53. doi: 10.1186/s13052-022-01236-3 (PMC8973802; doi:10.1186/s13052-022-01236-3)
Supplement: Supplementary file 1 — Additional file 1: Supplementary Table 1. Associations of obesity indices with elevated BP by univariate logistic regressions. [file 13052_2022_1236_MOESM1_ESM.docx]

Supplementary table 1. Associations of obesity indices with elevated BP by univariate logistic regressions.

| Variable | Male |  | Female |  |
| --- | --- | --- | --- | --- |
|  | OR（95% CI） | P | OR（95% CI） | P |
| ZBMI | 1.238（1.116, 1.373） | <0.001 | 1.184（0.960,1.461） | 0.114 |
| ZWC | 1.374（1.198, 1.575） | <0.001 | 1.145（0.941,1.394） | 0.176 |
| ZWHtR | 1.230（1.085, 1.396） | 0.001 | 1.073（0.911,1.264） | 0.400 |
| BMI Category^a^ | 2.666（1.469, 4.838） | 0.001 | 1.709（0.794,3.678） | 0.171 |
| WC Category^b^ | 4.379（2.189, 8.759） | <0.001 | 2.970（1.153, 7.650） | 0.024 |
| WHtR Category^c^ | 2.696（1.496, 4.858） | 0.001 | 1.371(0.642,2.928) | 0.414 |

^a^BMI category: overweight/ obese verses healthy.

^b^WC category: WC ≥ P_80_ versus WC < P_80_.

^c^WHtR category: WhtR ≥ 0.5 versus WHtR < 0.5.

All models were adjusted by age and amount of physical activity.
